# Recovery of male DNA acquired from carrion‐feeding insects in a simulated sexual assault scenario

**DOI:** 10.1111/1556-4029.70341

**Published:** 2026-04-24

**Authors:** Tinotenda Angel Mupfumi, Meenu Ghai, Danisile Tembe

**Affiliations:** ^1^ School of Agriculture and Science, College of Agriculture, Engineering and Science University of KwaZulu‐Natal Durban South Africa

**Keywords:** crop content, decomposition, forensic entomology, genetic markers, insect succession, polymerase chain reaction and PCR, quantitative polymerase chain reaction and qPCR, sexual assault, Y‐STR DNA

## Abstract

When bodies of sexual assault victims are discovered in advanced stages of decomposition, the recovery of direct DNA evidence is often compromised, posing a challenge in criminal investigations. This study aimed to determine insect colonization and succession patterns on pig carcasses inoculated with semen and to assess the feasibility of recovering Y‐chromosomal short tandem repeat (Y‐STR) DNA from fly larvae, fly pupae, and beetles. Three female pigs were used as models to simulate sexual homicide scenarios, and an indoor control experiment using chicken livers mixed with 3 mL of semen was also conducted. Larvae and pupae were collected throughout the decomposition process, with crop/gut contents dissected for DNA extraction using the phenol–chloroform method. DNA concentrations were quantified by qPCR, and Y‐STR profiling was attempted using the Yfiler™ Plus kit. In the field experiment, colonization by species from the families Calliphoridae, Sarcophagidae, Muscidae, Silphidae, Dermestidae, and Cleridae followed typical succession patterns, although arrival times varied between carcasses, with higher activity observed on semen‐inoculated pigs. Larvae from the indoor experiment yielded quantifiable male DNA and produced complete Y‐STR profiles at early time points (24–48 h), with partial profiles thereafter (72–96 h). In contrast, larvae from the field experiment contained only 1–3 pg. of male DNA, insufficient for profiling. These findings demonstrate that insect crop contents can preserve male DNA and occasionally yield usable Y‐STR data; however, DNA quantities are often too low for successful profiling. Future studies should therefore incorporate additional genetic markers alongside conventional Y‐STRs to enable profiling from degraded DNA.

**Highlights**

- Semen‐inoculated carcasses showed higher and earlier insect colonization than the control.
- Insect crop contents preserved male DNA from decomposing remains.
- Male DNA was successfully recovered from fly larvae.
- Complete Y‐STR profiles were generated from the fly larvae fed on liver tissue.
- Insect‐derived male DNA provided a reliable alternative source of male DNA for STR analysis.

## 1 INTRODUCTION

Studies have shown that one in every fourteen women has been a victim of sexual abuse at least once during her lifetime [1]. Globally, women are sexually assaulted daily, with little to no success in apprehending the perpetrator(s), highlighting the severity of the issue as a significant societal and forensic challenge [2, 3, 4]. The highest incidence of rape has been reported in countries such as South Africa, Botswana, India, Sweden, New Zealand, Canada, Zimbabwe, Brazil, and the United States, with South Africa ranking the highest globally [5, 6, 7]. A study by Coetzer [5] reported that 41% of rape victims in South Africa are often murdered following the sexual assault. In 2019 and 2020, the South African Police Services (SAPS) recorded approximately 1,919,495 violent cases, of which 53,293 were sexual offences from April 2019 to March 2020 [8]. Furthermore, from 2022 to 2023, similar trends of sexual offences persisted, with an alarming average of 70 murder cases per day across the country [9]. According to Naidoo [6], SAPS statistics revealed that only one in 20 rape cases are reported, of which 6.25% progress to official investigations [5]. This highlights the underreporting of sexual assault crimes relative to other criminal offences, which may be due to victims' fear that perpetrators may evade justice since few of these cases go to court [5, 10]. Surprisingly, Jewkes & Abrahams [10] reported that of all rape cases that go to court, only 7%–13% of the cases result in successful convictions in South Africa.

In most cases, the bodies of these rape victims are dumped in places that are difficult to locate; thus, sometimes, their bodies are discovered in advanced or skeletonized stages of decomposition [1, 11, 12, 13]. In such instances, critical physical evidence, particularly semen, is often degraded or entirely absent, limiting its potential use for DNA analysis and forensic investigation [12, 13, 14, 15, 16]. As such, many of these cases remain unsolved due to limited evidence [5, 10, 17]. However, insects found on a decomposing body can serve as an alternative tool to prove whether an individual was sexually assaulted before their death [1, 11, 12, 13, 16, 18]. During sexual assault, semen is normally deposited in the victim's genital or anal regions [1, 12, 13, 19]. Fly larvae are commonly attracted to these regions in high numbers, and as they consume decomposing tissue, they ingest the semen present on the body, consequently preserving the evidence [1, 18, 20, 21, 22, 23]. Several studies highlight the forensic value of insect‐derived DNA when human tissue is highly degraded or absent [21, 24, 25, 26]. For instance, in a study of de Lourdes Chavez‐Briones et al. [25], DNA extracted from insect larvae collected from a severely burned and mutilated body produced a partial profile of the victim, enabling the victim's identification using the father's reference sample. Similarly, Li et al. [21] reported successful analyses of short tandem repeats (STRs) and mitochondrial DNA (mtDNA) from both human tissue and larval crop contents, which yielded complete profiles, demonstrating that fly larvae can provide a reliable alternative source of human DNA when conventional samples are compromised.

In developed countries, the collection and use of forensic entomology evidence is a standard practice during forensic investigation and is used in courts [27, 28, 29]. However, in many African countries, including South Africa, the use of entomological evidence is still debatable, suggesting a need for more studies to support its application and reliability in medicolegal investigations [30]. Additionally, with the increasing prevalence of sexual assault homicide cases in South Africa, urgent regional research is needed to strengthen sexual assault evidence and complement the current efforts by the government and investigators in solving sexual homicide cases in this region [5, 6, 7, 8]. This study, therefore, aimed to determine insect colonization and succession on pig carcasses inoculated with semen and to assess the feasibility of recovering Y‐STR DNA from fly larvae and fly pupae of forensically important flies and beetles in South Africa.

## 2 MATERIALS AND METHODS

### 2.1 Ethics statement

This study used animal subjects and adhered to the relevant ethical standards of the Animal Research Ethics Committee of the University of KwaZulu‐Natal (AREC/00006345/2023). Section 20 authorization was granted prior to ethics approval, in full compliance with the guidelines governing the care, handling, and use of animals in biomedical research in South Africa.

### 2.2 Controlled experiment

Before the field experiment, a controlled trial was conducted for protocol optimization to establish whether Y‐STR DNA could be detected in fly larvae. Fresh chicken liver (125 g) was used as bait to attract flies and as a substrate for female oviposition [31]. The liver was cut into small pieces and placed in a shallow plastic container inside a metal cage (100 cm × 50 cm × 50 cm) to protect it from predators while allowing free movement of arthropods. The liver was monitored four times daily, at 3‐h intervals. On day three of the experiment, eggs hatched, and first‐instar fly larvae were collected and transported to the laboratory, where they were reared on fresh chicken liver under continuous darkness at a controlled temperature of 25 ± 2°C until further analysis.

Twenty larvae were collected using sterile forceps and evenly divided into two clear containers (Treatment 1 and Treatment 2), with Treatment 2 serving as a replicate of Treatment 1. Each treatment contained pieces of chicken liver mixed with 1.5 mL of semen, and larvae were allowed to feed. At 24, 48, 72, and 96 h post‐transfer, four larvae were collected at each time point and pooled to form a single sample, resulting in a total of four samples (*N* = 4). The samples were preserved in 70% ethanol until further analysis. At 24 h, larvae consisted of a mixture of first and second instar larvae. After 48 h, a mixture of second and third instars was identified; however, at 72 and 96 h, samples consisted entirely of third instars, and these were identified using size and morphological characteristics [32, 33]. Collected samples were washed with distilled water and homogenized to optimize DNA recovery. DNA was extracted using a standard phenol–chloroform protocol and eluted in 50 μL of TE buffer. DNA concentrations were quantified using the Quantifiler® Trio kit (Applied Biosystems, USA) and subsequently amplified with the Yfiler™ Plus kit (Applied Biosystems). The degradation index, which assesses DNA fragmentation by amplifying both short and large autosomal (LA) DNA targets, was calculated by the Real‐Time PCR software before STR analysis. Amplified fragments were separated by capillary electrophoresis on an ABI Prism® 3500 Genetic Analyzer, and the results were analyzed using Gene Mapper™ ID‐X software version 1.4.

### 2.3 Field experiment

**FIGURE 1:**
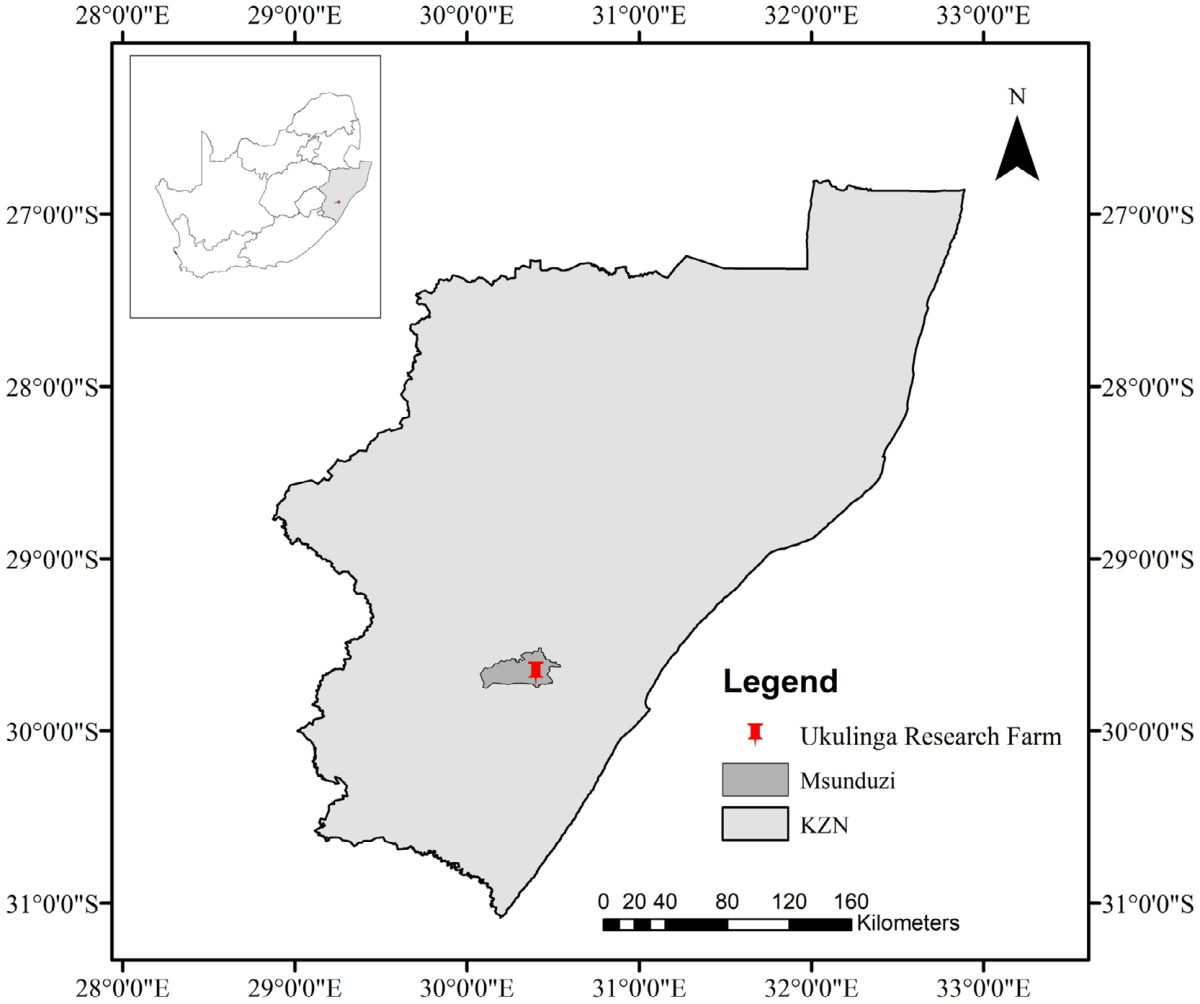
Map showing the study site in the uMgungundlovu District of KwaZulu‐Natal province.

The field experiment was conducted to assess the effectiveness of Y‐STR DNA recovery under conditions that simulate real crime scenes, specifically in cases where sexual assault victims are murdered and abandoned. The field experiments were conducted at the University of KwaZulu‐Natal, Ukulinga Research and Training Farm (29.6667° S, 30.40° E), located in Pietermaritzburg, South Africa (Figure 1). The study was conducted in 1 month (May 2024), with measured average atmospheric temperatures ranging from 24°C during the day to about 10°C at night.

### 2.4 Study animals, euthanasia of experimental animals, and semen inoculation

A total of three female domestic pigs (*Sus scrofa domesticus*), with an average live weight of 33 kg, were purchased from Misty Mount farm in Pietermaritzburg and used as human models to simulate sexual homicide scenarios. Following purchase, the pigs were transported to the Biomedical Research Unit (BRU) at the University of KwaZulu‐Natal, Westville campus, in Durban, and acclimatized for 3 days. The pigs were divided into two groups, with two pigs serving as experimental animals (Pig 1 and Pig 2) and one as a control (Pig 3).

After the acclimatization period, pigs were sedated with a combination of 2.21 mg of Medodin, 7 mg of Midazolam, and 7 mg of Butraphanol, administered through a 5 mL syringe with an 18‐gauge needle. To simulate male ejaculation, 3 mL of semen was inoculated into the vaginal and anal regions of the experimental pigs, with 1.5 mL administered to each region using a plastic syringe [13]. The control pig was inoculated with 3 mL of distilled water around the vagina and anus. Thereafter, all three pigs were euthanized using 10 mL of Barbiturates. The carcasses were immediately transported to Ukulinga Research Farm and placed in individual rectangular metal cages (100 cm × 50 cm × 50 cm) within a 20‐meter distance from each other. The metal cages were covered with mesh wire to protect the carcasses from predators while allowing unrestricted access for flies and other arthropods [34]. This study did not simulate and/or focus on bruises and traumas associated with sexual assault.

### 2.5 Insect sampling procedures and morphological observations of carcasses and insect activities

The first day on which animals were placed in the cages was recorded as day zero for all data collection, and the experiments lasted for 31 days. Postmortem visual body changes were monitored and recorded daily throughout decomposition and categorized into different stages of decomposition following the criteria outlined by Wolff et al. [35], Kyerematen et al. [36], and Comstock et al. [37]. Insect colonization and succession patterns between carcasses were observed and recorded throughout the study. Beetles, together with fly larvae and pupae, were collected from the anal and vaginal regions and surrounding soil using sterile forceps. Furthermore, adult arthropods found on and around the carcasses were collected using pitfall traps (containing glycerol), fly traps, and by direct hand‐picking using forceps [34, 38]. Abundance was categorized as low (<5 individuals), moderate (5–50 individuals), and high (>50 individuals) [15]. Flies and beetles were killed with ethyl acetate, and larvae and pupae were preserved in 70% ethanol until further processing.

### 2.6 Morphological identification of adult arthropods

Flies and beetles were soaked individually in 50 mL of distilled water for approximately 15 min, according to the date of collection. This was done to remove contaminants [13, 34]. The specimens were then air‐dried and grouped into different genera using a stereomicroscope and the identification keys described by Iqbal et al. [39] and Lutz et al. [40].

### 2.7 Dissection of fly larvae, pupae and beetles

Larvae collected from the controlled experiment and larvae, pupae, and adult beetles collected from the pig carcasses (field experiment) were individually cleaned with 50 mL of distilled water, air‐dried, and arranged per day and area of collection prior to dissection. Larvae and pupae were placed dorsally in the middle of a Petri dish, which was positioned under the stereomicroscope. Using a sharp blade, a longitudinal incision was made from the posterior to the anterior region [1, 13]. The blade was held at a low angle, and light pressure was applied, sufficient to pierce the cuticle (for larvae) or the puparium (for pupae) without damaging the internal organs. After making the incision, internal organs were exposed, and fine forceps were used to gently extract the gut from the surrounding tissue. First‐instar larvae were homogenized, as their small crops were extremely difficult to dissect. Second‐ and third‐instar larvae were dissected, and their gut contents were processed for subsequent DNA analysis. Older pupae with a hardened puparium could not be dissected. For adult beetles, the wings, head, and thorax were removed to allow direct access to the abdomen. A shallow dorsal incision was made along the abdominal tergites using a scalpel, after which the cuticle was reflected and the gut gently extracted from the surrounding tissue using fine forceps [41].

In total, 92 samples (*N* = 92) were processed from each experimental pig carcass, including 60 larvae samples, 24 pupae samples, and 8 beetle samples, with multiple replicates collected from each day and sampling area. All equipment (e.g., tweezers, scalpels, and Petri dishes) was cleaned with 70% ethanol and distilled water between each dissection to avoid contamination [1, 13]. The remaining larval, pupal, and beetle viscera were stored in separate 1.5 mL Eppendorf tubes at −20°C.

### 2.8 DNA extraction protocol

Genomic DNA was extracted from the semen reference sample with the DNeasy® Blood and Tissue Kit (Cat. No. 69506) using a user‐developed protocol for semen following the manufacturer's instructions. DNA was extracted from larvae and pupae using the conventional phenol–chloroform protocol for soft tissue, with DNA eluted in 50 μL of TE buffer. DNA quantity and purity were measured using a Nanodrop spectrophotometer (ND‐2000, Thermo Scientific, USA). Semen DNA, as well as larvae and pupae samples with DNA concentrations ranging from 50 ng/μL and above, were subjected to qPCR analysis.

### 2.9 DNA quantification, degradation, and Y‐STR typing

Y‐DNA quantification was performed using a Quantifiler® Trio DNA Quantification kit (Applied Biosystems, USA) in a 7500 Real‐Time PCR System (Applied Biosystems™, USA). The DNA degradation index (DI) was obtained from the quantified samples and served as an indicator of the extent of degradation prior to STR analysis [42]. The DI was calculated automatically by the Real‐Time PCR software using the formula: $\frac{\text{Small autosomal target}\;\mathrm{DNA}\;\text{concentration}\;\left(\mathrm{ng}/\mathrm{\mu L}\right)}{\text{Large autosomal target}\;\mathrm{DNA}\;\text{concentration}\;\left(\mathrm{ng}/\mathrm{\mu L}\right)\;}$, where the LA target reflects the overall DNA quality in a given sample (presence of intact, longer fragments), and the small autosomal (SA) target represents the total male DNA quantity (shorter fragments) [43].

Quantified DNA was then amplified using a Yfiler™ Plus kit (Applied Biosystems) in a Veriti 9600 thermal cycler (Applied Biosystems™), where a total of 27 Y chromosome loci were tested: DYS19, DYS385a/b, DYS389I, DYS389II, DYS390, DYS391, DYS392, DYS393, DYS437, DYS438, DYS439, DYS448, DYS456, DYS458, DYS635, DYS576, DYS533, DYS481, DYS449, DYS460, DYS518, DYS570, DYS539, DYS557, YGATAH4, DYF387S1, and DYS427. Amplified fragments were separated by capillary electrophoresis on an ABI Prism® 3500 Genetic Analyzer, and the results were analyzed using Gene Mapper™ ID‐X software version 1.4 (Applied Biosystems™).

### 2.10 Molecular identification of arthropods

Molecular techniques were further used to complement morphological identification for collected insects. DNA was extracted from the wings and legs of adult flies and beetles using a DNeasy® Blood & Tissue Kit (QIAGEN) according to the manufacturer's instructions. Individual DNA was then amplified using cytochrome c oxidase subunit I (COI) forward, LCO1490 (5′‐GGTCAACAAATCATAAAGATATTGG‐3′), and reverse, HCO2198 (5′‐TAAACTTCAGGGTGACCAAAAAATCA‐3′) primers [44]. PCR amplification was performed in a standard 25 μL reaction mixture, each containing 2× PCR Master Mix (Thermo Scientific), 10 μM of forward and reverse primer, 20–50 ng of template DNA, and sterile water. PCR analysis was performed in a Veriti 9600 thermal cycler (Applied Biosystems™) under the following thermal conditions: 95°C for 7 min, followed by 35 cycles of 60 s at 95°C, 60 s at 55°C, 60 s at 72°C, and lastly, a final extension period of 7 min at 72°C [38].

A separate PCR analysis was performed for beetles using the mitochondrial primers (F: 5′‐CAGATCGAAATTTAAATACTTC‐3′) and (R: 5′‐GTATCAACATCTATTCCTAC‐3′) [38]. The reaction was carried out in a 25 μL mixture under the following cycling conditions: 94°C for 3 min, followed by 35 cycles of 30 s at 94°C, 30 s at 50°C, 30 s at 72°C, with a final extension at 72°C for 5 min [34]. Two to three microliters of PCR products were separated on a 1% agarose gel stained with SYBR™ Safe (Thermo Scientific) for 1 h at 80 volts. Amplified products, with band sizes of 720 bp for flies and 272 bp for beetles [38], were sent for Sanger sequencing at Inqaba Biotech Industries (Pty) Ltd. (Pretoria, South Africa). Raw sequences were edited using BioEdit and identified using the Basic Local Alignment Search Tool (BLAST) of the NCBI (National Centre for Biotechnology Information) by identifying their closest match on the database.

## 3 RESULTS

### 3.1 Insect colonization patterns on the control and inoculated pig carcasses

**FIGURE 2:**
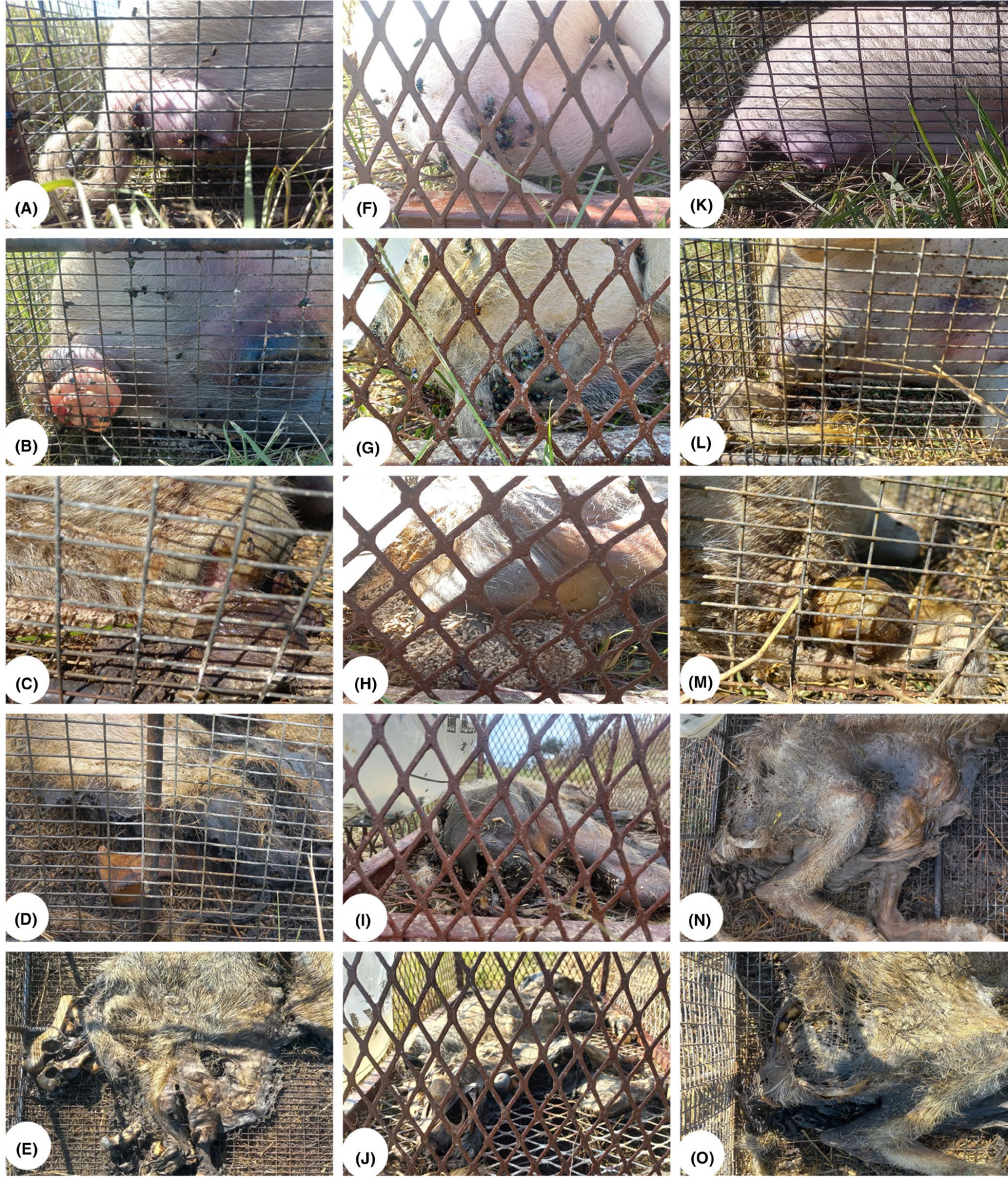
Photographs representing the effect of insect colonization around the genitals of inoculated (A–J) (Pigs 1 and 2) and control pig carcasses (K–O) (Pig 3). Images (A–O) illustrate the sequential decomposition stages throughout the experiment: (A, F, K) fresh stage (day 1), (B, G, L) bloated stage (day 5), (C, H, M) active decay stage (day 9), (D, I, N) advanced decay stage (day 17), (E, J, O) dry stage (day 31).

Postmortem changes in all pig carcasses were categorized into five decomposition stages: fresh, bloat, active decay, advanced decay, and dry (Figure 2). In the early stages, flies exhibited a strong preference for the semen‐inoculated carcasses (Pig 1 and Pig 2), particularly around the anal and vaginal regions. Adult flies, *Chrysomya albiceps* (Wiedemann) (Diptera: Calliphoridae), *Chrysomya marginalis* (Wiedemann) (Diptera: Calliphoridae), *Chrysomya putoria* (Wiedemann) (Diptera: Calliphoridae), *Chrysomya chloropyga* (Wiedemann) (Diptera: Calliphoridae), *Lucilia cuprina* (Wiedemann) (Diptera: Calliphoridae), *Musca domestica* (Walker) (Diptera: Muscidae), and *Sarcophaga calcifera* (Boettcher) (Diptera: Sarcophagidae), and beetles, *Thanatophilus micans* (Fabricius) (Coleoptera: Silphidae), *Dermestes maculatus* (De Geer) (Coleoptera: Dermestidae), and *Necrobia rufipes* (De Geer) (Coleoptera: Cleridae), collected during the study were confirmed to species level using DNA barcoding.

### 3.2 Fresh stage (0–1 day)

This stage commenced directly after death, with minimal physical and putrefactive changes (Figure 2). Adult flies were observed almost instantly, with *C. albiceps* and *M. domestica* being the first to arrive on the anal and vaginal regions of the semen‐inoculated carcasses. These were followed by *C. marginalis*, *C. putoria*, *C. chloropyga*, *L. cuprina*, and *S. calcifera*. In contrast, these species arrived approximately a day later on the control carcass (Table 1), where they were observed feeding on the mouth, nose, and eyes. Oviposition by *C. albiceps* and *L. cuprina* on treated carcasses began within 24 h, predominantly in the anal and vaginal regions, whereas on the control carcass, egg‐laying was delayed by 12–16 h and occurred mostly around the mouth, nose, and eyes. No larvae were observed during this stage.

**TABLE 1:** Overall abundance and succession patterns of insects at different stages of decomposition on inoculated and control pig carcasses.

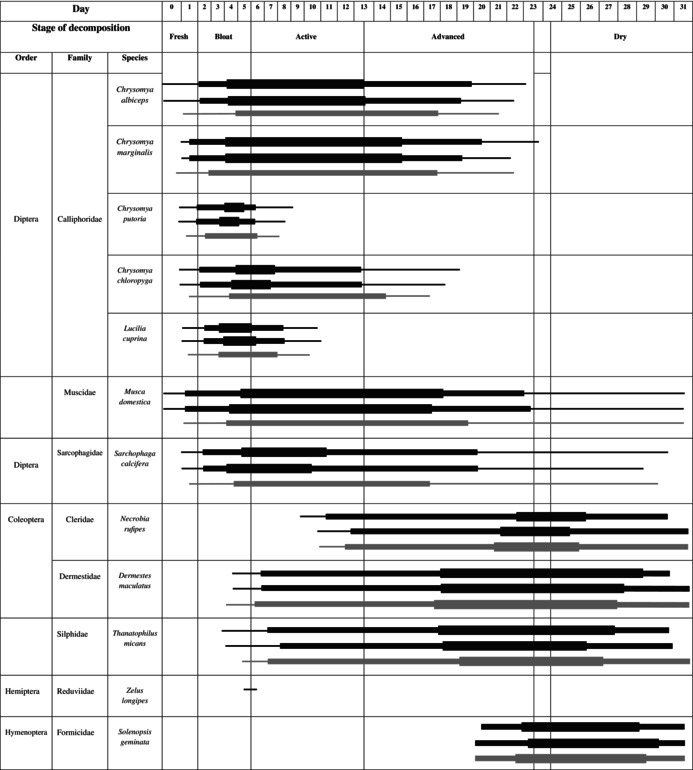

*Note*: Black fill – represents treated carcasses; Gray fill – represents the untreated carcass. The thickness of each arrow represents the abundance of insects. 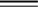 – Low abundance; 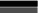 – Moderate abundance; 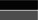 – High abundance.

### 3.3 Bloat stage (2–5 days)

At this stage, both the treated and untreated carcasses emitted a foul odour and were visibly swollen. On the treated carcasses, adult flies (*C. albiceps, C. marginalis, C. putoria, C. chloropyga, S. calcifera, M. domestica*, and *L. cuprina*) were predominantly concentrated around the bleeding anal and vaginal regions, with some fly activity on the abdomen and mouth (Figure 2B,G). On the control carcass, these species primarily fed and oviposited in the mouth, nose, and eyes, with limited activity in the anal and vaginal areas. First‐instar larvae of these species were observed following similar spatial patterns, particularly around the oviposition sites. *T. micans* and *D. maculatus* were the earliest beetle species observed on both carcass types (Table 1). Additionally, *Zelus longipes* (Linnaeus) (Hemiptera: Reduviidae) was observed toward the end of this stage on one of the treated carcasses but was not collected due to its lower numbers.

### 3.4 Active decay stage (6–12 days)

This stage was marked by rapid tissue breakdown and soft tissue liquefaction, with an intensified odour of decay in both treated and control carcasses. The anal and vaginal regions of treated carcasses became increasingly exposed (Figure 2C,H), while remaining relatively intact in the control carcass (Figure 2M). Adult dipteran species from earlier stages remained present, except for *C. putoria* and *L. cuprina* (Table 1). Larvae were also observed, particularly around oviposition sites. Unlike in the previous stages, flies showed no clear preference for the anal and vaginal regions of treated carcasses; instead, they fed and oviposited in the eyes, mouth, nose, ears, and abdomen. On the control carcass, similar feeding and oviposition patterns persisted, although more flies were observed around the anus and vagina compared to earlier stages. Beetle species, *T. micans* and *D. maculatus*, were observed throughout this stage in both the control and treated carcasses (Table 1). However, an additional beetle species, *N. rufipes*, was first observed in this stage for all three carcasses.

### 3.5 Advanced decay stage (13–22 days)

The odour of decay diminished, and tissue breakdown slowed in both the control and treated carcasses. Bones, teeth, and internal organs continued to disintegrate, increasing skeletal exposure in all carcasses (Figure 2). Moreover, the abundance of *C. albiceps, C. marginalis, C. chloropyga*, *M. domestica*, and *S. calcifera* declined as this stage progressed. Although both adult flies and larval activity persisted on both carcass types, it decreased overall, with most larvae migrating underground to pupate. Beetle activity became more prominent, with *D. maculatus*, *N. rufipes*, and *T. micans* numbers increasing and persisting throughout this stage (Table 1). Additionally, *Solenopsis geminata* (Fabricius) (Hymenoptera: Formicidae) was observed toward the end of this stage on all carcasses.

### 3.6 Dry stage (23–31 days)

This stage was characterized by less smell, brittle bones, and dry skin in all carcasses. Additionally, the treated carcasses had pelvic bones protruding near the anal and vaginal regions (Figure 2E,J). The dipteran species, *M. domestica* and *S. calcifera*, along with *S. geminata*, persisted throughout this stage in both the control and treated carcasses with multiple pupal stages and no records of larval activity. In addition to dipteran species, *D. maculatus*, *N. rufipes*, and *T. micans* persisted on this stage and were actively feeding on the dry remains (Table 1).

### 3.7 Quantitation of male DNA

Male DNA was successfully quantified in all the larvae samples that fed on the liver mixed with semen. Male DNA concentrations were measured at 1.907 ng/μL, 1.266 ng/μL, 1.121 ng/μL, and 1.00 ng/μL at 24, 48, 72, and 96 h, respectively, with an average concentration of 1.32 ng/μL. In the larvae samples collected from the vaginal and anal regions of experimental Pig 2 on days 4 and 5, male DNA was detected at low concentrations: 0.003 ng/μL (3 pg) on day 4 and 0.0011 ng/μL (1.1 pg) on day 5. An additional sample collected near these regions on day 5 yielded a concentration of 0.001 ng/μL (1 pg). No quantifiable male DNA was detected in larvae samples from days 6 to 14, pupae from days 10 to 22, or in any larvae/pupae samples from pig 1 across all collection days. Therefore, these samples were excluded from further analysis. Moreover, the LA target for the quantified samples from pig 2 was undetermined, precluding the calculation of the DI. No male DNA was quantified in all the beetles that were collected.

### 3.8 Y‐STR genotyping

**FIGURE 3:**
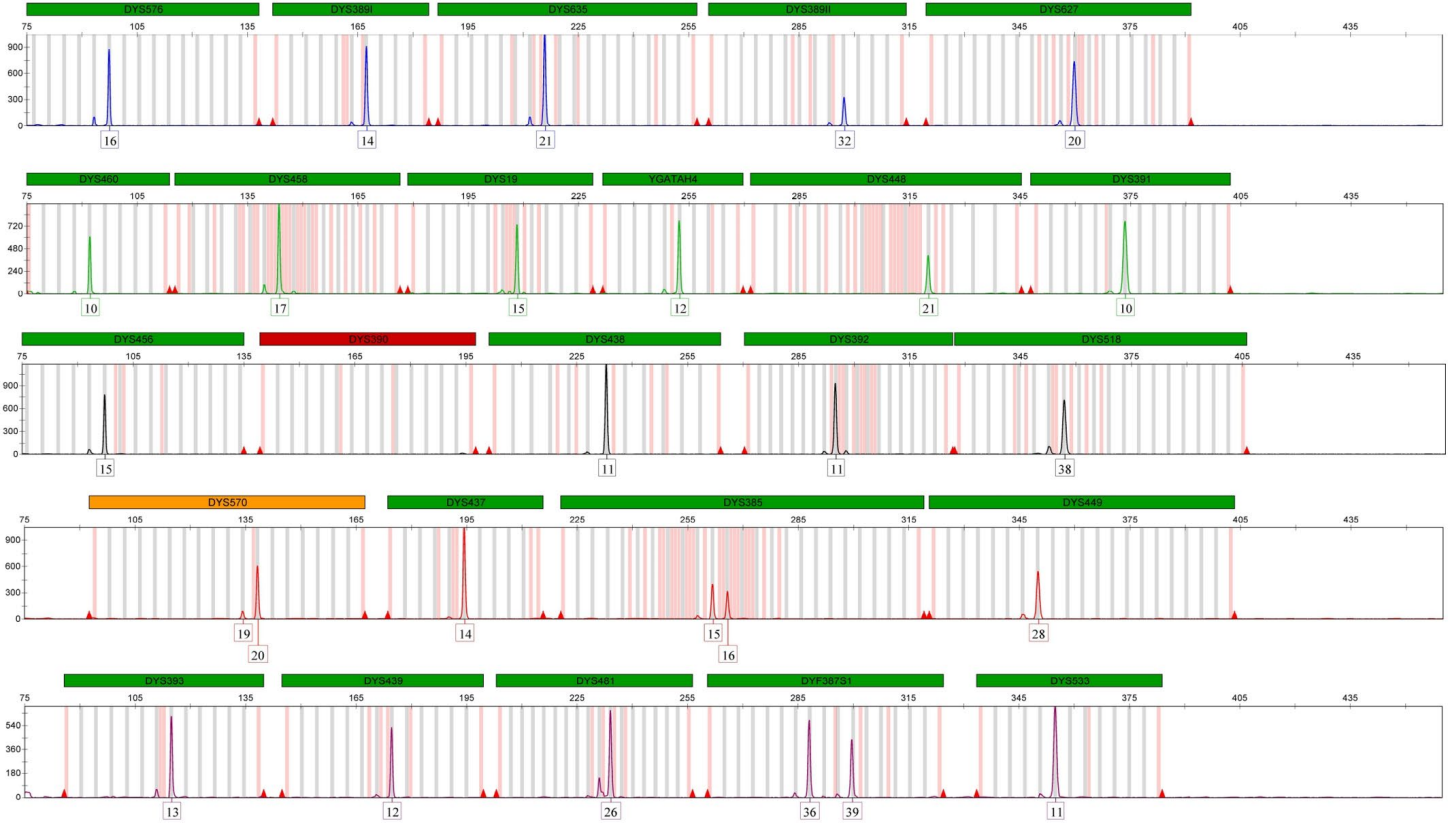
Y‐STR profile of the semen reference sample.

**FIGURE 4:**
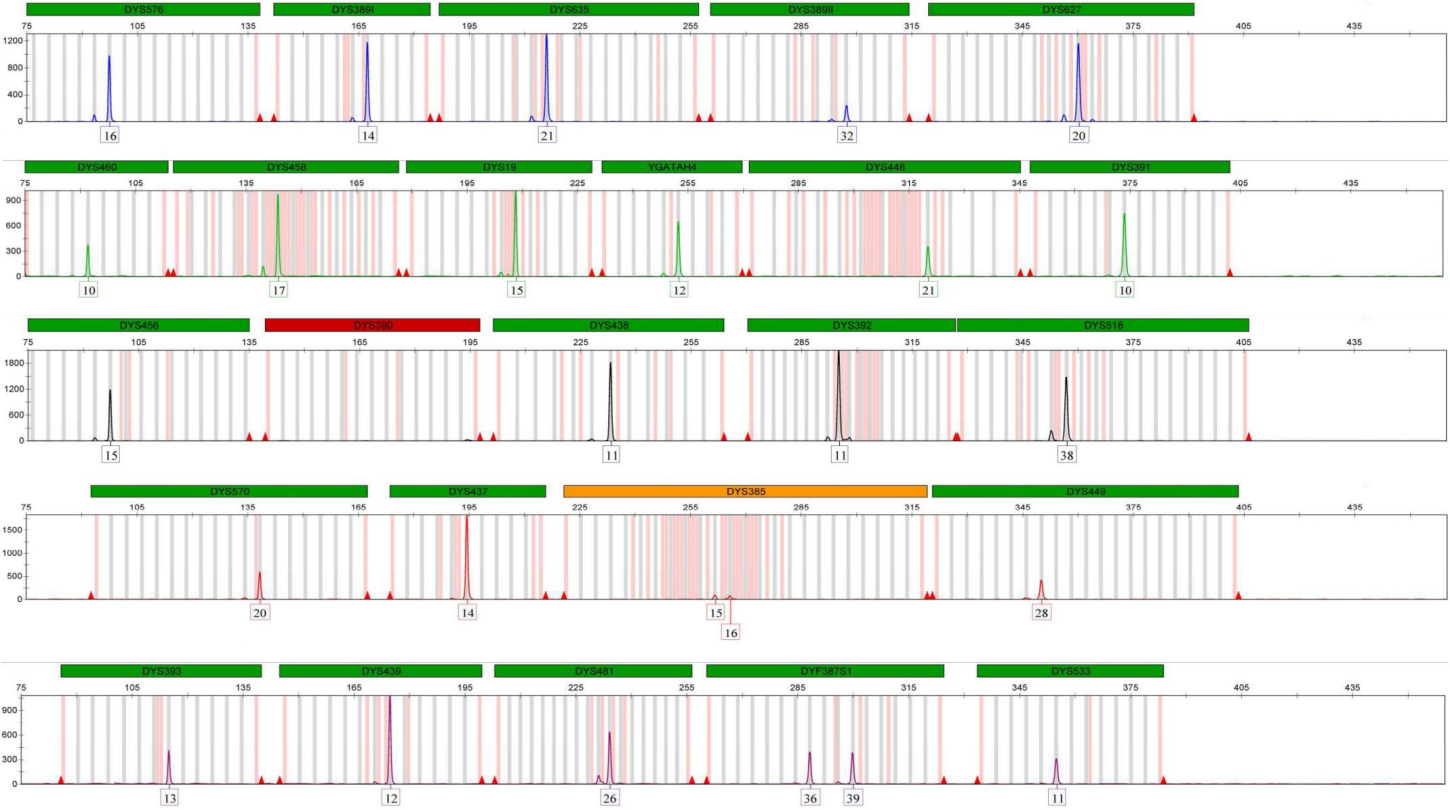
Y‐STR profile of male DNA extracted from maggots after 24 h of feeding on chicken liver.

**FIGURE 5:**
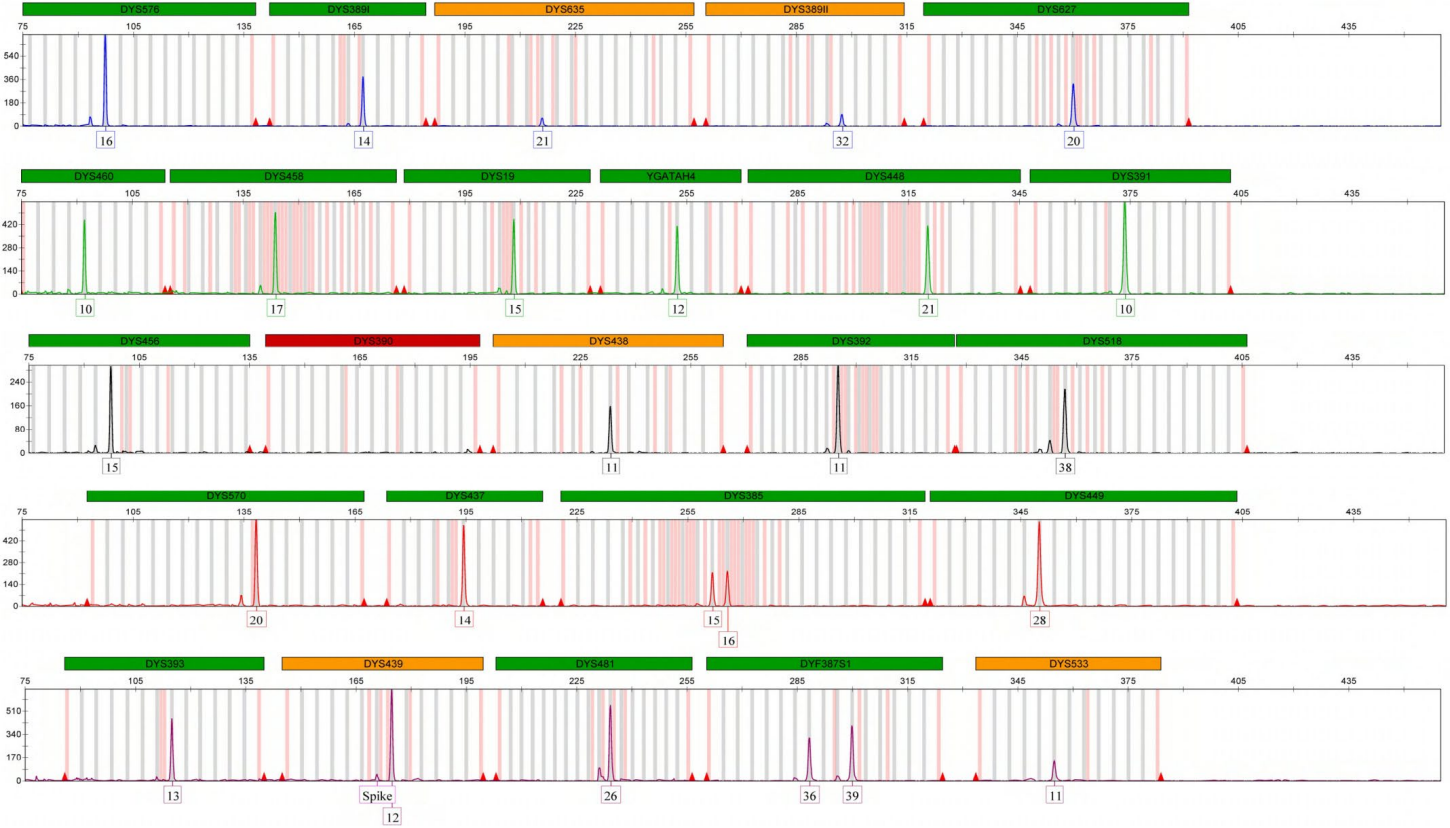
Y‐STR profile of male DNA extracted from maggots after 48 h of feeding on chicken liver.

**FIGURE 6:**
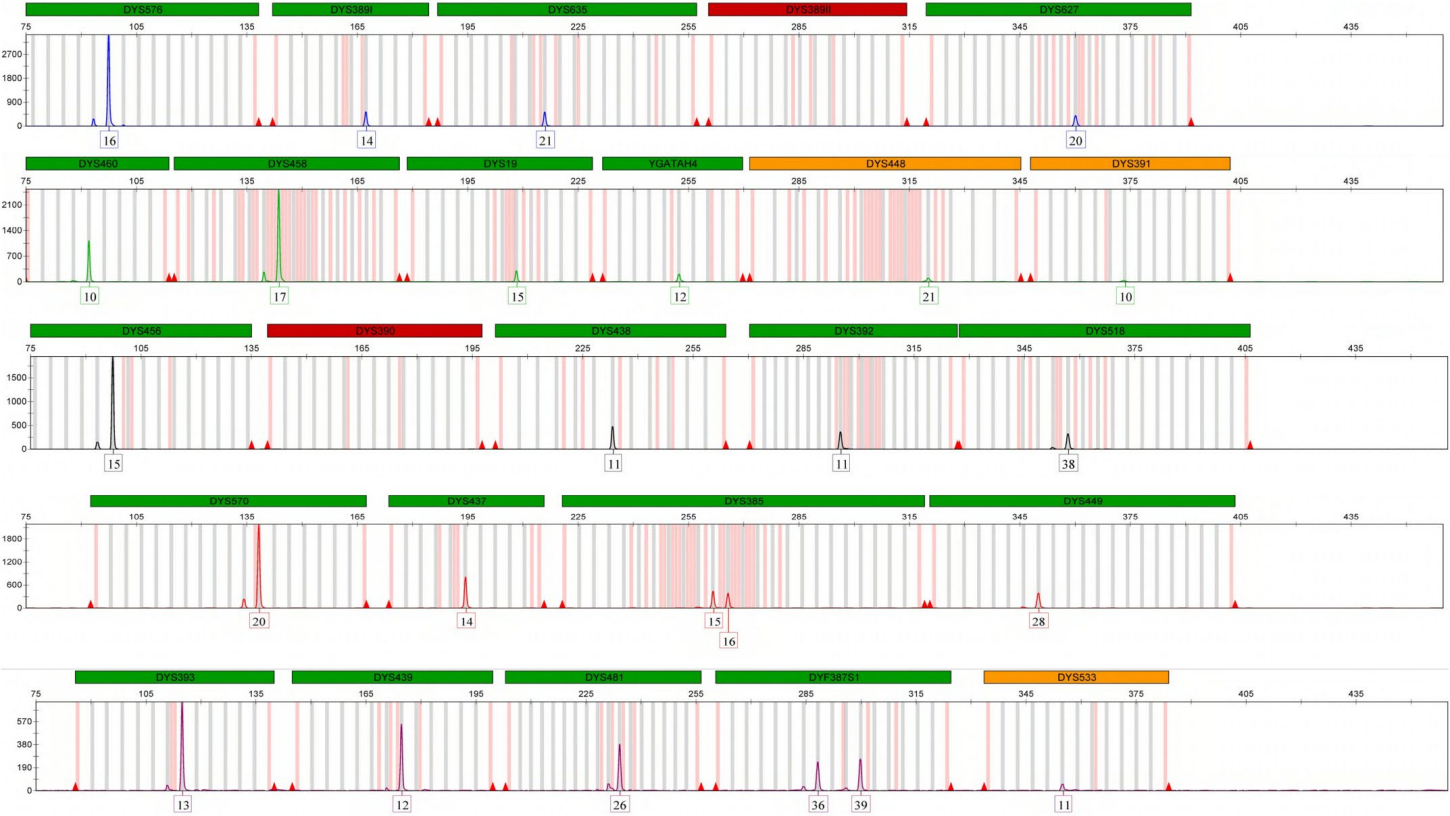
Y‐STR profile of male DNA extracted from maggots after 72 h of feeding on chicken liver.

**FIGURE 7:**
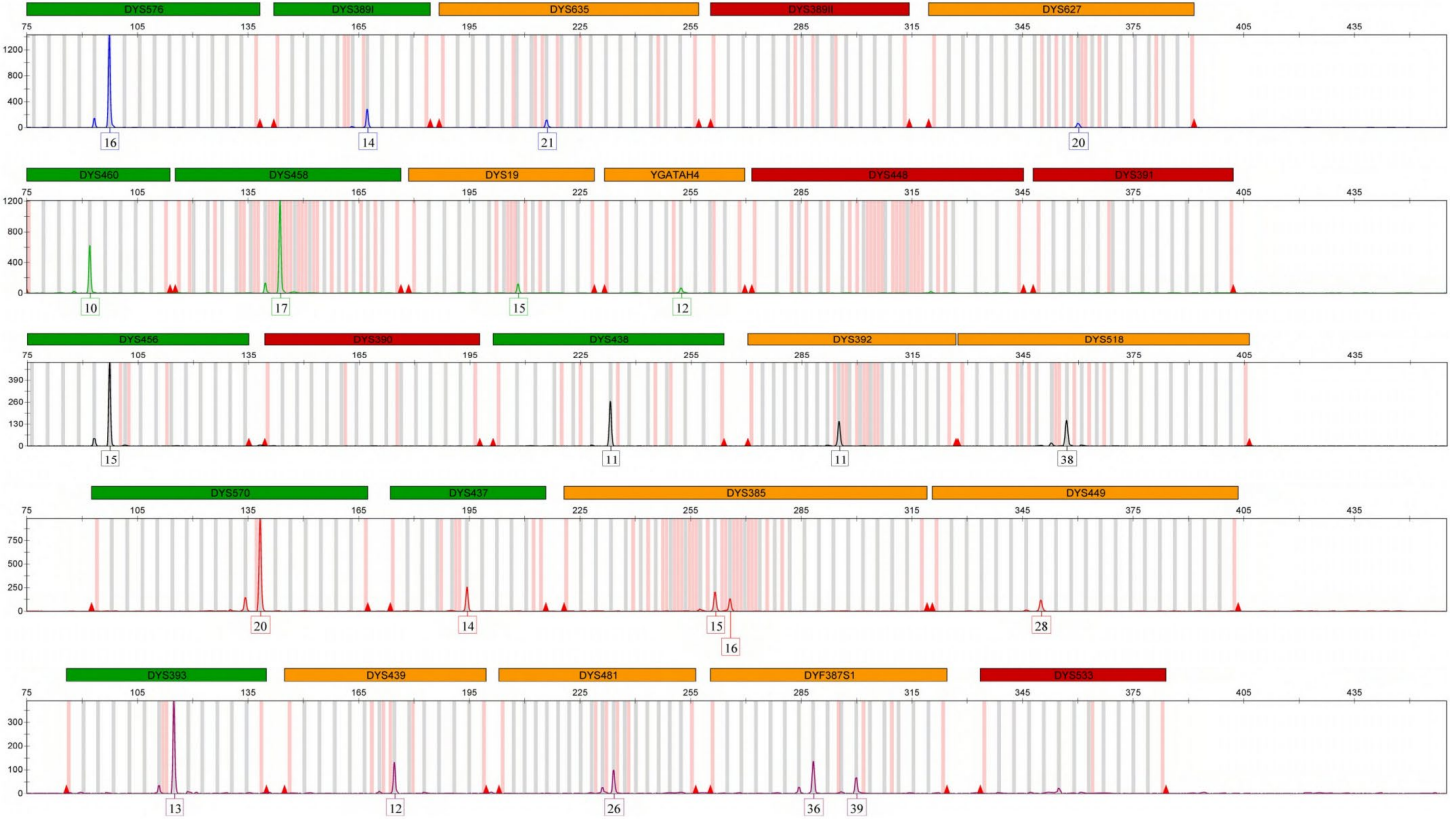
Y‐STR profile of male DNA extracted from maggots after 96 h of feeding on chicken liver.

Y‐STR genotyping for the semen reference sample was successful, although DYS390 displayed null alleles across all samples (Table 2; Figure 3). All loci (except DYS390) were amplified in the Y‐STR profiles generated for larvae samples collected from the liver at 24 (Figure 4) and 48 h (Figure 5). However, by 72 h, allele dropouts occurred at DYS389II and DYS627 (Figure 6), and by 96 h, additional dropouts were observed at DYS448, DYS391, and DYS533 (Figure 7). Furthermore, from 48 h onwards, the electropherograms displayed a ski‐slope effect, characterized by progressively lower peak heights and reduced amplification efficiency over time. Longer amplicons were more susceptible to this decline than shorter amplicons. No Y‐STR profiles were generated for the larvae samples collected on days 4 and 5 from experimental pig 2, despite successful quantification.

**TABLE 2:** Y‐STR profile of the reference semen sample and male DNA extracted from larvae samples feeding on liver (controlled experiment) collected at 24 h, 48 h, 72 h, and 96 h.

STR marker	Decomposition time and the amplified alleles					
	C1 (24 h)	C2 (48 h)	C3 (72 h)	C4 (96 h)	Combined genotype	Reference genotype
DYS576	16	16	16	16	16	16
DYS3891	14	14	14	14	14	14
DYS635	21	21	21	21	21	21
DYS389II	32	32	–	–	32	32
DYS627	20	20	–	–	20	20
DYS460	10	10	10	10	10	10
DYS458	17	17	17	17	17	17
DYS19	15	15	15	15	15	15
YGATAH4	12	12	12	12	12	12
DYS448	21	21	21	–	21	21
DYS391	10	10	10	–	10	10
DYS456	15	15	15	15	15	15
DYS390	–	–	–	–	–	–
DYS438	11	11	11	11	11	11
DYS392	11	11	11	11	11	11
DYS518	38	38	38	38	38	38
DYS570	20	20	20	20	20	20
DYS437	14	14	14	14	14	14
DYS385a/b	15/16	15/16	15/16	15/16	15/16	15/16
DYS449	28	28	28	28	28	28
DYS393	13	13	13	13	13	13
DYS439	12	12	12	12	12	12
DYS481	26	26	26	26	26	26
DYF387S1	36/39	36/39	36/39	36/39	36/39	36/39
DYS533	11	11	11	11	11	11

*Note*: Dash (–): Not amplified.

## 4 DISCUSSION

The decomposition stages observed in the control and treated carcasses were consistent with those described by Goff [11], Tembe & Mukaratirwa [34], and Sharanowski et al. [45], who utilized pig carcasses in their studies. Sequence analysis of the mitochondrial COI gene successfully identified the insects to species level as *C. albiceps*, *C. marginalis*, *C. putoria*, *C. chloropyga, L. cuprina*, *M. domestica*, *S. calcifera, T. micans*, *D. maculatus*, and *N. rufipes*, confirming morphological identification. From these, Dipteran families Calliphoridae (*C. albiceps, C. marginalis, C. putoria, C. chloropyga*, and *L. cuprina*), Sarcophagidae (*S. calcifera*), and Muscidae (*M. domestica*) were the dominant early colonizers on both the control and treated carcasses. These results align with the findings by Kolver [15], Tembe & Mukaratirwa [34], Kelly [46], and Tembe et al. [47]. Colonization initially occurred much faster on the semen‐treated carcasses compared to the control, although this difference became less pronounced at later stages of decomposition. This may be attributed to the presence of semen and its components, fucose, fructose, glucose, and disaccharides, which attract and stimulate flies and provide energy, making it an ideal feed for dipteran species in early decomposition [12, 48, 49]. Similarly, Durdle et al. [48] demonstrated that both 1‐day‐old and 3‐day‐old female blowflies (*L. sericata*) reared from the same cohort exhibited a strong preference for semen over other human biological fluids, such as blood and saliva. This preference is reflected in the 12–16‐h delay in oviposition in the control carcass, compared to its occurrence within 24 h in the treated carcasses, highlighting the influence of semen on insect oviposition site selection [48, 50]. During the bloated stage, blowflies (*C. albiceps*, *C. marginalis*, *C. putoria*, *C. chloropyga*, *S. calcifera*, *M. domestica*, and *L. cuprina*), known to feed more efficiently on liquids [12], fed intensely on the anal and vaginal regions of the treated carcasses, causing bleeding, which further attracted oviposition by fly colonies. In contrast, on the control carcass, fly activity was concentrated around the mouth, nose, and eyes, following a normal, predictable colonization pattern [11, 15, 51, 52]. The presence of Coleoptera species, *T. micans* and *D. maculatus*, on both untreated and treated carcasses during this stage aligns with findings by Tembe & Mukaratirwa [34], Mayer & Vasconcelos [53], and Singh & Bala [54].

The active decay stage was characterized by dipteran species that were present from the onset of decomposition on both treated and control carcasses. However, *C. putoria* and *L. cuprina* began to decline towards the end of this stage as signs of desiccation became evident [15, 38, 47, 55]. Although fly preference for the vaginal and anal regions of the treated carcasses diminished at this stage, larval masses remained concentrated in these areas due to initial oviposition and feeding preferences. Comstock et al. [37], Campobasso et al. [56], and LeBlanc & Logan [57] observed that while Coleoptera species may appear earlier in decomposition, their numbers typically increase during active decay due to the continued breakdown of soft tissue, which provides a more favourable environment for beetle activity. A similar trend was observed in this study, with populations of *T. micans, D. maculatus*, and *N. rufipes* increasing on both treated and control carcasses. In the advanced stage of decay, species of *C. albiceps, C. marginalis, C. chloropyga, M. domestica*, and *S. calcifera* declined significantly in all three carcasses due to the limited availability of moist tissue, as female Diptera do not lay eggs on dry remains [11, 15, 34, 51]. As fly activity decreased, *D. maculatus*, *N. rufipes*, and *T. micans* became more dominant, as beetles are better adapted to feeding on dry tissues [15, 34, 46, 58]. Moreover, the increase of predatory beetles (*N. rufipes* and *T. micans*) feeding on fly larvae likely reduced dipteran emergence [11, 34, 52]. Ants from the family Formicidae, particularly *S. geminata*, were observed in large numbers during both the advanced and dry stages, likely due to the availability of residual organic materials and reduced competition from other insects [59, 60]. By the dry stage, the physical impact of flies' preference for semen treated carcasses in the earlier stages of decay became more evident as these carcasses had pelvic bones protruding near the anal and vaginal regions, while the control had none. Notably, only *M. domestica* and *S. calcifera* persisted to this stage as the remaining tissue was not optimal for egg‐laying, reflecting patterns reported by Tembe et al. [38, 47], Horenstein et al. [58], and Magni et al. [61]. *D. maculatus*, *N. rufipes*, and *T. micans* were observed throughout this stage across all carcasses, actively feeding on the dry remains [15, 34, 51, 62].

Complete Y‐STR profiles were obtained from larvae fed on semen‐treated liver after 24 and 48 h, with partial profiles observed at 72 and 96 h due to progressive DNA degradation. These findings are consistent with those reported by Clery [18], who successfully recovered Y‐STR profiles from fly larvae, and with studies by Njau et al. [63] and Oliveira et al. [64], who reported full profiles within 48 h and partial profiles thereafter using autosomal STRs, with Oliveira et al. [64] additionally targeting X‐STRs. Loci dropout at 72 and 96 h began with larger amplicons (DYS389II, DYS627, DYS448, DYS391, and DYS533), as they are more susceptible to degradation due to their longer sequence length and increased vulnerability to strand breaks under environmental stressors such as heat, moisture, and enzymatic activity [65, 66, 67]. In a related study, Chamoun et al. [1] recovered Y‐STR DNA from the gastrointestinal contents of *C. albiceps* larvae fed on 600 g of ground beef mixed with 3 mL of male semen. Combined larvae samples amplified 15 of the 16 tested loci after 8 days; however, individual samples showed inconsistent allele dropouts, with 7 loci amplifying on day 5 and 12 on day 8, suggesting variations in primer hybridization efficiency. In contrast, our results showed that when a locus dropped out on a given day, it did not reappear in subsequent analyses. Notably, DYS390 failed to amplify across all samples, including the semen reference sample. This is likely due to allele‐specific polymorphisms in the primer binding region, which can result in null or silent alleles even in high‐quality DNA [68, 69]. Alternatively, it may represent a true null allele resulting from deletion of the entire locus caused by structural rearrangements within the Y‐chromosome azoospermia factor regions (AZFa, AZFb, and AZFc) [70]. In addition, Zieger & Utz [71] observed kit‐dependent dropout of DYS390 in their Swiss dataset, with some Y‐STR multiplexes (e.g., Yfiler Plus™) failing to amplify the locus while others succeeded. Nevertheless, true null alleles at DYS390 appear to be extremely rare, with the Y Chromosome Haplotype Reference Database (YHRD) documenting only a small number of such observations [72].

Male DNA was successfully recovered from the gut contents of larvae collected on days 4 and 5 from experimental Pig 2; however, no genetic profiles could be generated due to the extremely low DNA quantities. According to Clarke et al. [17], it is not always guaranteed that larvae that have consumed semen will be collected during sampling and contain sufficient DNA, as multiple larvae masses often infest the same region, which could have been the case in our study. Increasing the number of samples collected is therefore recommended to improve the likelihood of recovering and amplifying target DNA. Additionally, semen degradation due to environmental exposure [73, 74] and/or biochemical alterations in the larvae's crop during digestion may have further compromised DNA quality [75]. Similarly, a study by Chamoun et al. [13], simulating a sexual crime using pigs inoculated with 3 mL of human semen, demonstrated a similar degradation pattern, with an average of one or two loci being amplified daily from days 6 to 14 of the 14‐day decomposition period, despite collection starting on day 4. While controlled experiments often produce reliable results, there remains a critical need for more field‐based studies that reflect real‐world forensic scenarios.

Vernarecci et al. [67] suggested that concentrating degraded samples with DNA concentrations of approximately 500 pg. (0.5 ng) or lower could yield full profiles. However, in the present study, attempts to concentrate male DNA from field samples collected on days 4 and 5 were unsuccessful. Specifically, larvae collected from the vaginal and anal regions had concentrations of 3 pg. (day 4) and 1.1 pg. (day 5), while larvae collected near these regions on day 5 yielded 1 pg. All three values fell well below the 500 pg. amplification threshold of the Yfiler™ Plus kit, likely explaining the failure to produce genetic profiles [76, 77]. While 500 pg. is considered the ideal threshold for obtaining a reliable Y‐STR profile, Haarkotter et al. [77] reported that as little as 5 pg. of male DNA can yield partial profiles in approximately 50% of cases. Nonetheless, concentrations below this typically fail to amplify, making 5 pg. a practical sensitivity limit for obtaining a detectable profile [63, 77]. While Njau et al. [63] successfully amplified human DNA from four‐day‐starved larvae with a concentration of less than 50 pg. using the AmpFLSTR™ Identifiler Plus kit, the resulting STR profile had very low peak heights. This raises concerns about the reliability of DNA profiles from degraded or low‐concentration samples in real‐world forensic investigations, where high‐quality profiles with distinct peak heights [75] are critical for accurate perpetrator identification. Haarkotter et al. [77] demonstrated that the PowerPlex® Y23 kit successfully generated complete Y‐STR profiles for victim identification from degraded skeletal remains buried in mass graves for 70–80 years, despite the high female DNA background. Complete profiles were obtained from samples with 62.5 pg. of male DNA in a mixture with 40,000 pg. of female DNA or 125 pg. of male DNA in a background of 3,000,000 pg. of female DNA. However, in sexual assault homicides, where the body is abandoned, male DNA from the perpetrator is often present in lower amounts [78, 79], below the amplification threshold required for reliable amplification. This highlights the limitations of current Y‐STR kits and emphasizes the need for kits with even lower detection thresholds, given the global prevalence of sexual assault.

In instances where conventional Y‐STR typing fails due to extensive DNA degradation or extremely low template quantities, alternative genotyping strategies may provide enhanced analytical success [75, 80, 81]. Although relatively few studies have examined the recovery of human DNA from dipteran larvae, existing research indicates that the success of downstream processing for any forensic sample is dependent on whether appropriate extraction methods are applied [80, 81]. For example, Cantu et al. [81] demonstrated improved recovery of human DNA from fly larvae using the DNeasy® PowerSoil® Pro kit, which incorporates Inhibitor Removal Technology (IRT) to neutralize humic acids and chitin that typically inhibit PCR amplification in fly larvae DNA extracts. Furthermore, the study showed that mtDNA analysis and Next‐Generation Sequencing (NGS) can provide investigative leads when conventional STR typing fails. In addition, mini‐STR systems, which target shorter amplicons, are particularly well suited for degraded samples, as they can reduce locus dropout by improving amplification efficiency in fragmented DNA [81, 82, 83, 84, 85]. The integration of single‐nucleotide polymorphisms (SNPs) offers an additional advantage for highly degraded larval samples, as these markers can often be successfully genotyped even when STR profiles cannot be obtained [75, 80, 81]. Li et al. [86] further demonstrated that combining Y‐STR and Y‐SNP markers into a dual‐marker system improves resolution and analytical precision by targeting complementary male‐specific regions of the Y chromosome. Although additional genotyping of the pig carcass samples was beyond the scope of this study, future research should incorporate additional genetic markers alongside conventional Y‐STRs to enhance sensitivity and reliability in challenging forensic samples.

## 5 CONCLUSION

This study demonstrated that insect activity on decomposing carcasses can serve as a potential indicator of sexual assault, as fly species showed increased attraction to semen‐inoculated regions, particularly the vaginal and anal regions. Male‐specific DNA was successfully recovered from the gut contents of larvae collected from these areas, supporting the use of carrion‐feeding insects as alternative sources of male DNA in sexual assault investigations in this region. These overall results contribute to forensic entomology research and can be used as a baseline in solving suspected sexual assault cases where bodies are recovered in different stages of decomposition. The authors recommend future studies on optimizing protocols for the recovery and amplification of very low‐template DNA from carrion‐feeding insect samples.

## FUNDING INFORMATION

National Research Foundation, (South Africa), Thuthuka grant No. TTK2204052012.

## CONFLICT OF INTEREST STATEMENT

The authors have no conflicts of interest to declare.

## Data Availability

The data that support the findings of this study are available from the corresponding author upon reasonable request.
